# Exploring the Influence of NaOH Catalyst on the Durability of Liquid Calcium Aluminate Cement Concrete

**DOI:** 10.3390/ma18153655

**Published:** 2025-08-04

**Authors:** Chung-Lin Lin, Chia-Jung Tsai, Leila Fazeldehkordi, Wen-Shinn Shyu, Chih-Wei Lu, Jin-Chen Hsu

**Affiliations:** 1 Department of Civil Engineering, National Pingtung University of Science and Technology, Pingtung City 912301, Taiwan; s205900@gmail.com (C.-L.L.); wsshyu@mail.npust.edu.tw (W.-S.S.); 2 Department of Civil and Construction Engineering, National Taiwan University of Science and Technology, Taipei 106335, Taiwan; cwlu@mail.ntust.edu.tw; 3 Department of Mechanical Engineering, National Yunlin University of Science and Technology, Douliu City 64002, Taiwan; hsujc@yuntech.edu.tw

**Keywords:** alkali–aggregate reaction, NaOH, liquid calcium aluminate cement

## Abstract

Liquid calcium aluminate cement (LCAC) is an innovative material technology with significant potential for varied applications in civil engineering. However, despite its promising results, a significant gap remains in the direct application of LCAC as a concrete binder. The primary catalysts for LCAC are sodium hydroxide (NaOH) and potassium hydroxide (KOH). Therefore, it is crucial to investigate the effects of sodium and potassium ions on alkali–aggregate reactions in concrete structures. This study evaluated the durability of liquid calcium aluminate cement concrete catalyzed using four different concentrations of NaOH (0.5%, 1.0%, 1.5%, and 2.0%) as experimental variables, incorporating a control group of traditional concrete with a water–cement ratio of 0.64. The findings indicate that NaOH catalysis in the concrete significantly trigger alkali–aggregate reactions, leading to volume expansion. Furthermore, it increased chloride ion penetration and porosity in the concrete. These effects were more notable with the increase in NaOH concentration. The results suggested that NaOH catalysis can enhance certain chemical reactions within the concrete matrix; however, its concentration must be carefully controlled to mitigate adverse effects. The NaOH dosage should be limited to 0.5% to ensure optimal durability of the concrete. This study emphasizes the crucial importance of precisely balancing catalyst concentration to maintain the long-term durability and performance of liquid calcium aluminate cement concrete in structural applications.

## 1. Introduction

Rapid economic growth has led to substantial development, resulting in a significant increase in the demand for construction materials to upgrade infrastructure. Consequently, industrial sectors have expanded over decades, and their environmental impacts related to carbon dioxide (CO_2_) emissions have become a global concern. The construction industry is responsible for nearly 3% of the total CO_2_ emissions, which are emitted by the cement clinker production process [1,2]. Moreover, it is one of the largest industrial sectors in terms of natural resource utilization and energy consumption. The sustainable development agenda aims to achieve long-term goals, particularly the reduction in carbon dioxide emissions [3]. Therefore, optimizing the cement process has become the center of attention to achieve the scenario of net-zero emissions by 2050 [1,4,5]. Recent advancements in sustainable cement technologies have focused on reducing the carbon footprint of cement production, particularly through alternative binders and low-clinker formulations [5,6]. Calcium aluminate cement (CAC) has garnered attention due to its lower CO_2_ emissions during manufacturing, faster setting times, and superior resistance to aggressive environments [7,8,9]. However, its use remains limited primarily to specialized applications such as repairs, refractory materials, or corrosion-resistant coatings [10]. In addition, liquid calcium aluminate cement (LCAC), referred to as Suspended Cement (SC), offers promising flexibility and durability enhancements due to its delayed hydration properties. CAC and SC are high-alumina cements, which differ by their form, properties, uses, and advantages. SC is a form of CAC where the cement is delivered in a liquid suspension instead of dry powder. Therefore, CAC requires water for its hydration and setting process, while SC is a pre-hydrated liquid or slurry that requires an alkaline solution or component [11,12,13]. Although both offer similar benefits, such as rapid compressive strength, hardening properties, thermal resilience, and corrosion resistance [14,15,16,17], SC offers more advantages over classical CAC due to its smaller particle size, higher reactivity, and better hydration control [12]. LCAC mitigates constraints related to rapid setting and transportation difficulties inherent in powdered CAC [11]. Moreover, SC acts as a retarder to prevent the hydration of calcium aluminate cement. This is primarily achieved through the use of sodium gluconate [18] or other inorganic compounds such as phosphates, borates, and metal salts. Therefore, it can maintain a liquid state before activation, providing a more flexible application [19,20,21]. Calcium aluminate cement is produced by proportionally mixing and grinding limestone and alumina with a relatively high aluminum oxide content. The primary source of its reactivity is monocalcium aluminate (CA). In contrast, Calcium Silicates (C_2_S and C_3_S) are the primary sources of reactivity in traditional Portland Type I cement. Compared to C_2_S and C_3_S, the CA compound formation temperature and time are comparatively lower. Therefore, calcium aluminate cement emits less carbon dioxide during the manufacturing process than traditional Portland Type I cement [22]. Accordingly, calcium aluminate cement is considered a low-carbon, environmentally friendly cement. Currently, suspension cement is used in the construction and paint industries for decorative purposes, as flooring underlayment, for repairs, as insulation material [10], or as an additive that leverages the characteristics of aluminate cement to enhance abrasion resistance and erosion resistance in civil structures, such as spillways in dams and walkways [23]. It is especially effective in resisting the corrosion of acidic substances [24] and in applications requiring rapid hardening [25]. However, no comprehensive research has been conducted on the feasibility of its structural application. There is a lack of systematic evaluation of the durability and mechanical performance of SC when activated with various alkali catalysts. In addition, the impact of catalyst concentration on long-term concrete behavior and the risk of alkali–aggregate reaction (AAR) has not been sufficiently examined in the context of SC-based concretes. This study aims to fill this gap by assessing the potential application of suspension cement in concrete structures through standardized testing and comparing its performance to traditional Portland cement concrete.

Considering that SC is fluid, cement reactions require an alkaline catalyst, which results in solidification with water. Manufacturers currently recommend two types of catalysts: metal alkalis, NaOH and KOH. However, using Na^+^ and K^+^ ions may increase the risk of alkali–aggregate reaction (AAR) in concrete structures. The alkali–aggregate reaction is a mechanism for the degradation of aggregate mineral components and alkali metal ions Na and K, with alkali metal hydroxides dissolving in the concrete pore solution. The reaction product is a gel viscous substance with a hygroscopic nature that absorbs water molecules and expands. As a result, it causes swelling within the cementitious matrix. The main symptoms include staining and gel exudation on the concrete surface, structural displacement, and the development of irregular cracks arranged in a map-like pattern on the concrete surface [26,27,28,29]. Previous studies have demonstrated that alkali metal ions, particularly Na⁺ and K⁺, play a critical role in the hydration processes and microstructural development of high-alumina cement (HAC) systems, including liquid-phase variants such as LCAC. Lachowski et al. (2000) confirmed that the presence of Na⁺ and K⁺ modifies the formation pathways and stability of hydration products such as CAH_10_, C_2_AH_8_, and C_3_AH_6_, which influence the porosity and long-term volumetric stability of the material [30]. Blanco-Varela (2005) and Pastor et al. (2009) stated that alkali ions influence the arrangement and stability of aluminohydrate phases, consequently affecting ion permeability and durability performance [31,32]. In addition, Li et al. (2018) reported that under high-temperature conditions, alkali salts containing Na⁺ and K⁺ react with alumina-rich refractory materials and form low-melting potassium–sodium aluminate compounds, resulting in microstructural deterioration and diminished stability [33]. It is generally recommended that the alkali content (Na_2_O + 0.658K_2_O) not exceed 0.6%, as it may have a potential impact on the durability of concrete structures, such as alkali–aggregate reactions [34]. Furthermore, alkalis originating from aggregates have been identified as significant contributors to the degradation of calcium aluminate cement (CAC)-based concrete. Specifically, both alkaline hydrolysis and AAR are known to initiate at or near the concrete surface, leading to the formation of loosely bound bayerite deposits. These deposits compromise the integrity of the aggregate–matrix interface, thereby significantly reducing the mechanical strength of the material. Such deterioration has been observed in prestressed CAC beams, highlighting the susceptibility of high-alumina cement (HAC) systems to degradation in strongly alkaline environments [35]. Despite these findings, the underlying mechanisms responsible for such deterioration, particularly in modern low-calcium and suspension-type high-alumina cements (LCAC and SCAC), remain inadequately understood, especially under aggressive exposure conditions.

Therefore, this study applied NaOH with four different concentrations of 0.5%, 1.0%, 1.5%, and 2.0% by weight of the cementitious content to assess the catalyst impact on the durability of suspension cement concrete. Given the relationship between water and cementitious content in suspension cement, the water–cement ratio is estimated to be around 0.64. Hence, traditional concrete with a water–cement ratio of 0.64 is used in the control group. Durability tests, including the volume stability test, concrete resistivity test, and salt ponding test, were conducted to better understand the impact of NaOH catalyst on the durability of suspension cement concrete and to simultaneously compare its performance with that of traditional cement concrete.

## 2. Experimental

**Figure 1:**
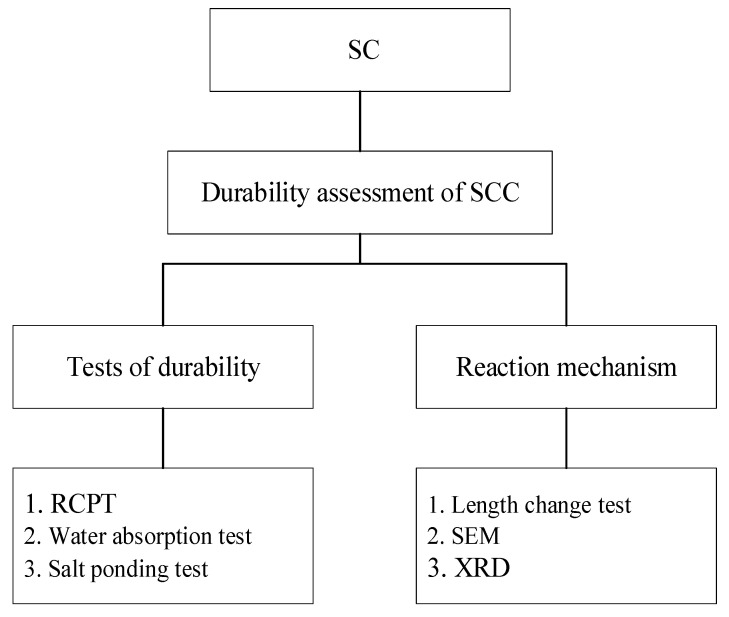
Research flowchart.

SC is recognized for its lower carbon footprint compared to conventional cement and its ease of use. However, its application as a binder in concrete structures raises durability concerns. The present study evaluated the durability performance of SC as a binder in concrete through a series of standardized experimental tests. The research methodology included multiple assessments to determine key durability parameters, including chloride ion penetration, dimensional stability, and microstructural properties. Specifically, six primary tests were conducted: the rapid chloride penetration test (RCPT), water absorption test, salt ponding test, length change test, scanning electron microscopy (SEM), and X-ray Diffraction (XRD). These tests were designed to assess the durability of SC-based concrete (SCC) and investigate the underlying reaction mechanisms. Figure 1 illustrates the methodology of this study. Furthermore, this study explored the impact of varying NaOH concentrations on the activation of SC and compared its performance with that of ordinary Portland cement (OPC) concrete. This study offers valuable insights into the potential of SC as a sustainable and durable alternative in concrete applications by thoroughly evaluating SCC under diverse conditions.

### 2.1. Mix Proportion

SC, with approximately 61% solid content, which corresponds to a water-to-cement ratio of about 0.64, was selected to investigate its durability as a bonding material in concrete structures. Considering that activation degree varies with different NaOH concentrations, the experiment was conducted using concentrations of 0.5%, 1.0%, 1.5%, and 2.0% by weight (*w*/*w*), which were labeled as SCN05, SCN10, SCN15, and SCN20, respectively. A minimum NaOH dosage of 0.5% was selected following the regulatory standard, which typically recommends that the total alkali content in cement (expressed as Na_2_O + 0.658K_2_O) should not exceed 0.6% [36]. This baseline dosage ensured compliance with accepted alkali limits while providing a reference point for assessing the potential risks of higher NaOH concentrations. Simultaneously, to compare SC concrete (SCC) and traditional concrete, ordinary Portland cement concrete (OPC) with a water-to-cement ratio of 0.64 was also selected as a reference group. Table 1 shows the detailed concrete mix proportions used in the experiments.

### 2.2. Materials

Liquid calcium aluminate cement (Imerys S.A., Paris, France): solid content of approximately 61%, viscosity of nearly 500 mPa·s, and a pH of about 6.31.

Fine aggregate: this study used natural river sand with a fineness modulus of 3.1, a specific gravity of 2.53, and a water absorption rate of 2% as the fine aggregate (Chaoqun, Pingtung, Taiwan).

Coarse aggregate: natural coarse aggregate (Chaoqun, Pingtung, Taiwan) with a maximum particle size of about 20 mm, a dry-rodded unit weight of 1654 kg/m^3^, a specific gravity of 2.61, and a water absorption rate of 0.68% is used.

Catalyst: the activator in this study is high-purity NaOH with a purity of approximately 95% (ECHO CHEMICAL Co., Ltd., Miaoli, Taiwan).

### 2.3. Methods

This study employs a comprehensive experimental approach to investigate the durability of Suspended Cement (SC) as a bonding material in concrete structures. The research method integrates multiple standardized tests to evaluate key performance indicators, such as chloride ion penetration, length change, and microstructural properties. The methodology is designed to assess the effects of different NaOH concentrations on the activation of SC and to compare the performance of SC concrete (SCC) with ordinary Portland cement concrete (OPC). The research methodology comprises four main experimental tests: the salt ponding test, length change test, rapid chloride penetration test (RCPT), and microstructure analysis. Each test was carefully selected to address specific aspects of concrete durability, such as resistance to chloride ion penetration, dimensional stability, and structural integrity at the micro-level. These tests were conducted following standardized protocols (AASHTO T259, ASTM C157-75, ASTM C1202-19, and ASTM C114-18) to ensure the reliability and comparability of the results [37,38,39,40]. For each test, three specimens were prepared for every OPC and SCC mix. In the salt ponding test, measurements were taken at three depth intervals (0–1 cm, 1–2 cm, and 2–3 cm), in a total of 15 specimens. The length changes were measured at 1, 3, 7, 14, and 28 days using 15 specimens, and rapid chloride penetration tests (RCPT) were conducted using 15 specimens to ensure robust statistical analysis and adequate representation. Results were reported as the mean of independent measurements, and the error bars in the figures indicate the standard deviation, reflecting the variability among replicates and supporting the reliability and transparency of the experimental data. This study aims to provide a detailed evaluation of the performance of SC concrete under various conditions, contributing to the understanding of its potential use in concrete structures.

#### 2.3.1. Salt Ponding Test

The salt ponding test, under the AASHTO T259 standard, involves drying the sample surface, followed by sealing its sides with epoxy resin. After the epoxy resin had solidified to ensure a tight bond and avoid water leakage from the sides, silicon resin was applied between a plastic ring and the specimen. Following drying, the top was filled with a 3% sodium chloride solution, and the plastic ring was sealed with plastic wrap. In the next step, the specimen was placed in a ventilated room with a temperature of 23 ± 5 °C. Throughout the 90-day test period, the liquid level at the top of the specimen was periodically checked, and the solution was added or replaced as needed. After 90 days, powder was extracted from a specific depth on the top surface of the specimen to measure chloride ion concentrations at various depths. During sampling, the plastic ring around the specimen was removed, and any remaining salt crystal residues on the surface were gently brushed away after the specimen had dried. Samples at various depths were obtained by coring and extracted using an acid dissolution method following the ASTM C114-18 standard for the chemical analysis of hydraulic cement. For dispersion, 10 g of ground powder were placed in a 250 mL beaker with 75 mL of deionized water; also, for acidification, 25 mL of dilute nitric acid was added. After covering the beaker and heating it to a boiling point, the solution was cooled to room temperature and then filtered through filter paper to determine the concentration of chloride ions.

#### 2.3.2. Length Change Test

This experiment adhered to the specifications outlined in ASTM C157-75, “Standard Test Method for Length Change of Hardened Cement Mortar and Concrete”. Following casting and molding, specimens underwent moist curing within the molds for 23.5 ± 0.5 h to assess the length variations of the SCC. Then, they were demolded and immersed in water for 24 h, and their lengths were measured. The initial lengths were used as a reference. The specimens were kept in water at a temperature of 23 ± 1 °C for curing. Additionally, their lengths were measured at 1, 3, 7, 14, and 28 days. The length change ratio is calculated by Equation (1).

(1) $$\mathrm{Length}\;\mathrm{change}\;(\%)=\frac{A-B}{A}\times 100$$

where *A* represents the measured length after 24 h of immersion in water (mm), and *B* is the measured length at various ages (mm).

#### 2.3.3. Rapid Chloride Penetration Test (RCPT)

**Figure 2:**
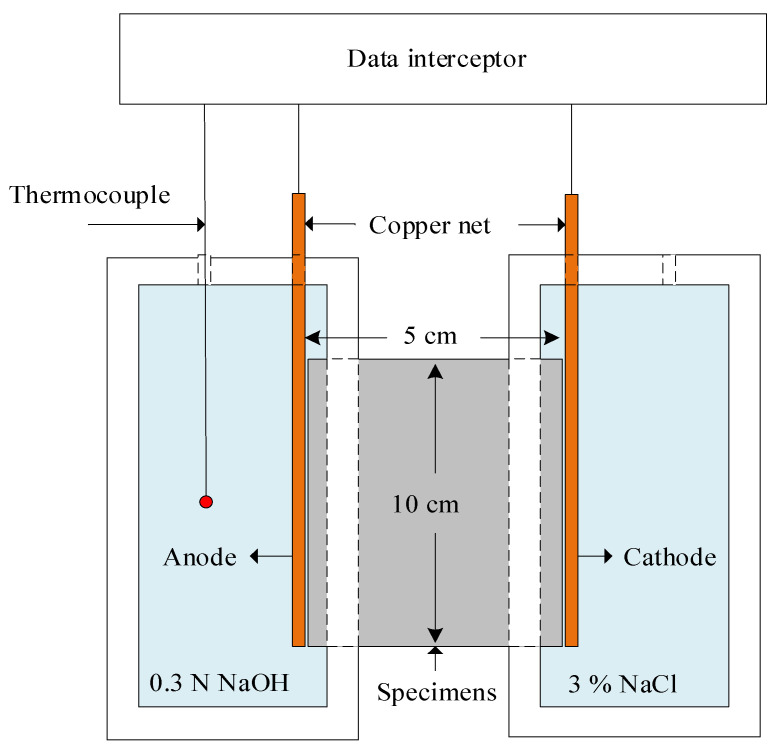
Configuration of rapid chloride penetration test.

This experiment followed the standard test method ASTM C1202–19, “Standard Test Method for Electrical Indication of Concrete’s Ability to Resist Chloride Ion Penetration”. After 28 days of moist curing, the cylindrical specimens were extracted, and the central portions were cut with a cutting machine. In the next step, two specimens with lateral dimensions (D) of 10 cm and longitudinal dimensions (H) of 5 cm were selected for the rapid chloride ion penetration test. The cut specimens were dried in an oven (100 ± 5 °C) and placed at room temperature for 24 h. Afterward, waterproof material was applied around the specimens and placed in a vacuum chamber. The vacuum pump was used to achieve a vacuum level of 1 mmHg atmospheric air pressure and kept there for 3 h. The stop valve was opened, allowing distilled water (cooled) to flow into the vacuum chamber and cover the specimens. The vacuum pump is then reopened to continue evacuating internal air (a step lasting at least one hour). The vacuum pump was then switched off, and the vacuum chamber was opened to allow air to enter. The specimens were taken out and immersed in water for 18 ± 2 h. Finally, the specimens were placed in an electrical permeability cell, with the cathode chamber containing a 3% sodium chloride solution and the anode chamber containing a 0.3 N sodium hydroxide solution, completing the entire electrical circuit configuration. A continuous DC voltage of 60 ± 0.1 V was applied for 6 h. The current was recorded every 30 min, and the total passed charge was calculated using the trapezoidal method with the following Formula (2). Figure 2 illustrates the rapid chloride penetration test configuration.

(2) $$Q=900(I_{00}+{2I}_{30}+{2I}_{60}+{\ldots \ldots \ldots ..+2I}_{300}+{2I}_{330}+I_{360}$$

In the equation, the variables are as follows:

Q represents the total passed charge in coulombs.

I_0_ represents the initial current value in amperes after applying an external voltage.

I_t_ represents the current value in amperes at time t minutes after applying an external voltage.

#### 2.3.4. Microstructure Analysis

A high-resolution field emission scanning electron microscope (FE-SEM) model S-3000N (Hitachi High-Tech Corporation, Tokyo, Japan) was employed to observe the microscopic structure and comprehend the changes in the microstructure of SCC response to the alkali–aggregate reaction. The magnification mechanism uses an electron beam emitted from a field emission source, which impacts the specimen, generating signals. These signals are amplified and delivered to a cathode ray tube, ultimately displaying images of microscopic structures on a screen.

The experiment also utilized an Energy Dispersive Spectroscopy (EDS) device (HORIBA, Ltd., Kyoto, Japan), which was attached to the SEM. The analysis principle involves a silicon (or lithium) detector operating under reverse bias. The emission of high-energy electron beam X-rays results in the formation of electron-hole pairs, which generate energy pulses. By applying an external voltage, the movement of electrons and holes produces energy pulsations. After that, a multichannel analyzer records the voltage pulsations to identify the peak values and provides the possibility to analyze the elemental composition of the material.

## 3. Test Results and Discussion

Table 2 lists the test results for four evaluations: RCPT, water absorption test, salt pond test, and length change test. Results include total charge (coulombs, C), water uptake (%), chloride content (%), and length change (%) through the RCPT. The total charge obtained through RCPT provides insight into the chloride ion permeability of SCC. This helps to understand the effect of NaOH on the durability of SCC and the difference compared to the durability of conventional concrete. In addition, salt pond testing is used to verify the consistency of test results by evaluating the penetration of the natural diffusion of chloride ions. Water absorption tests and length change tests were further used to explore potential factors affecting durability.

### 3.1. Rapid Chloride Penetration Test Results

**Figure 3:**
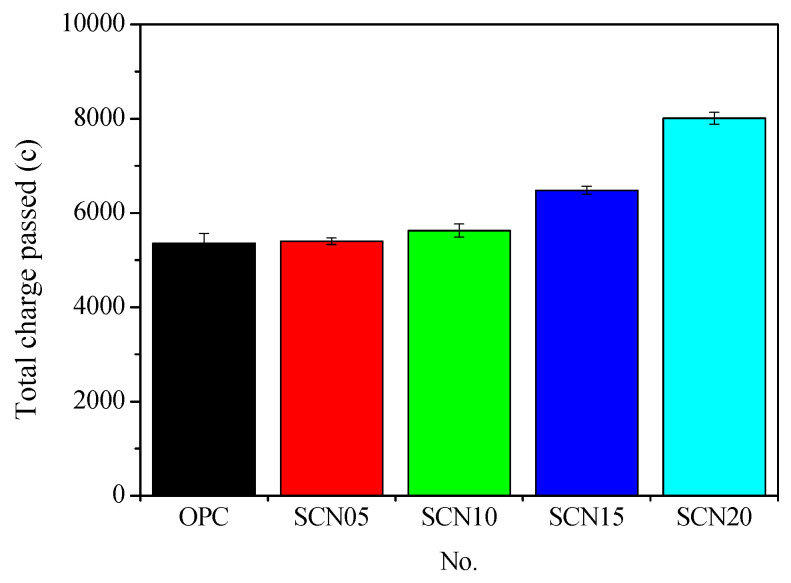
Comparison of the cumulative total charge passed between SCC and OPC.

Figure 3 represents the RCPT results for SCC and OPC. In NaOH concentrations of 5% (SCN05) and 10% (SCN10), the cumulative total charge transferred by SCC is closer to the value of ordinary Portland cement concrete (OPC). However, these numbers are still greater than OPC, at around 0.84% for SCN05 and 5.04% for SCN10. For SCN15 and SCN20, the values are relatively higher, showing increases of 21.01% and 49.58% compared to conventional concrete.

**Figure 4:**
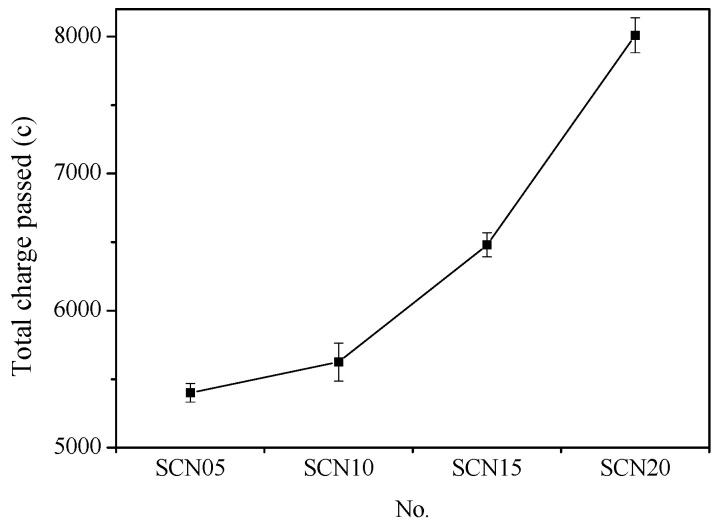
Relationship curve of cumulative total charge passed for SCC with different NaOH addition levels.

Figure 4 depicts the relationship between the cumulative total charge passed and the different NaOH dosages in SCC. The results showed that NaOH concentration increase leads to an increase in the total cumulative charge passed. In general, the optimal performance was observed at NaOH with a concentration of 0.5%, which showed a cumulative total charge similar to OPC. On the other hand, the rise in the cumulative total charge passed caused by NaOH concentration implies a decrease in SSC durability.

### 3.2. Salt Ponding Test Results

**Figure 5:**
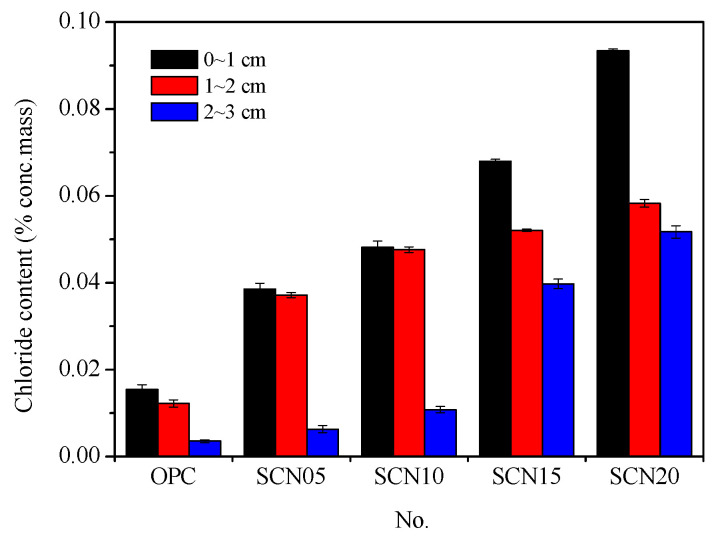
Comparison chart of chloride ion concentration between SCC and OPC.

The salt ponding test was used to measure the resistance of concrete to chloride ion penetration, which indicates its durability. This method is typically time-consuming and requires 90 days. After 90 days of immersion, slices of hardened concrete were collected at depths of 0–1 cm, 1–2 cm, and 2–3 cm, and the ratios of chloride ion concentration were determined. The results indicated that SCC catalyzed by NaOH has higher chloride ion concentrations than OPC at each layer. Among the SCC samples, the SCN05 showed the lowest increase in chloride content at the 2–3 cm depth, with approximately 1.74 times the concentration found in OPC, followed by SCN10 with an increase of nearly 2.99 times. In contrast, SCN15 and SCN20 showed higher concentrations, with increases of approximately 11.04 and 14.36 times, respectively (Figure 5).

**Figure 6:**
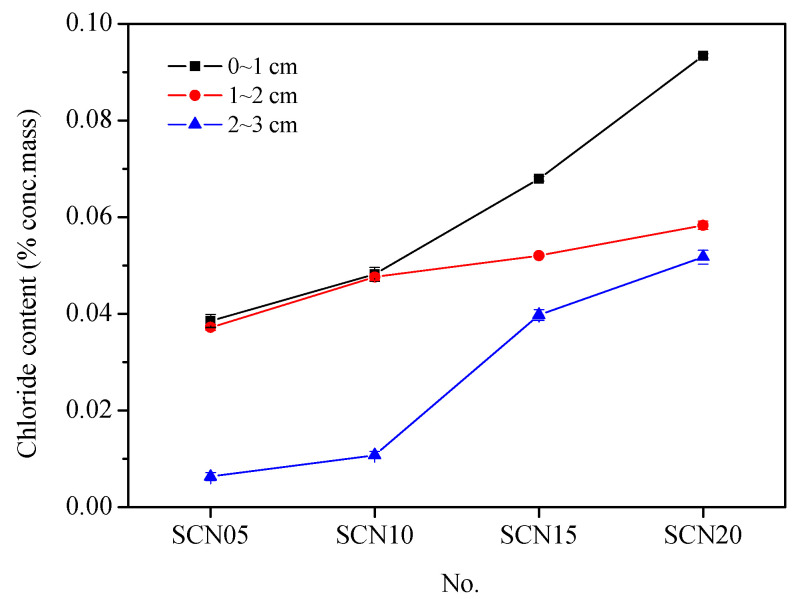
Chloride ion concentration profiles at different depths for SCC with various NaOH addition levels.

Figure 6 illustrates the chloride ion concentration curves at different depths for SCC with varying NaOH dosages. Overall, higher NaOH dosages result in higher chloride ion concentrations.

**Figure 7:**
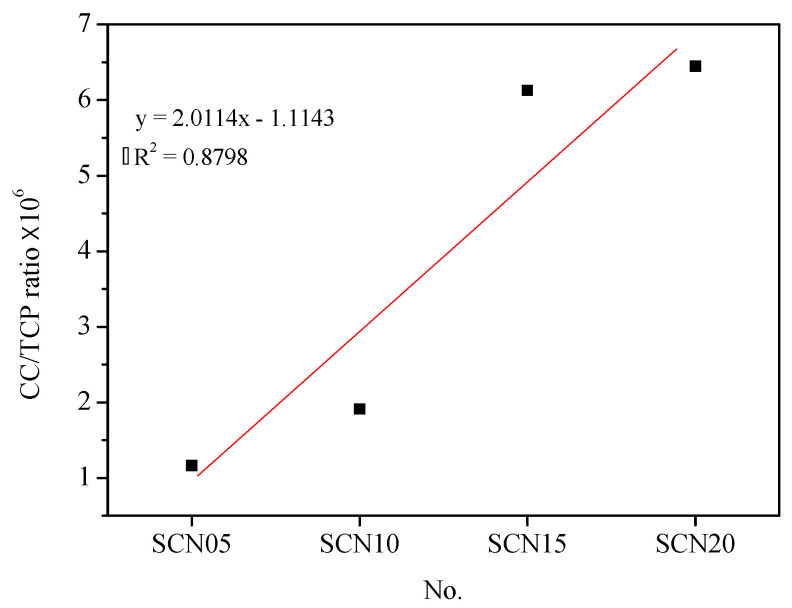
Trend chart of chloride ion concentration (CC) and cumulative total charge passed (TCP) in SCC.

However, chloride ion concentrations in SCN05 and SCN10 at 1–2 cm depth have shown a remarkable upward trend. In addition, a significant positive correlation (with an R_2_ value of 0.88) was found between chloride ion concentration (CC) and cumulative total charge passed (TCP), indicating that NaOH concentrations influence the durability of SCC. Consequently, higher NaOH dosages result in less durability (Figure 7).

### 3.3. Impact of Pores on the Durability of SC

**Figure 8:**
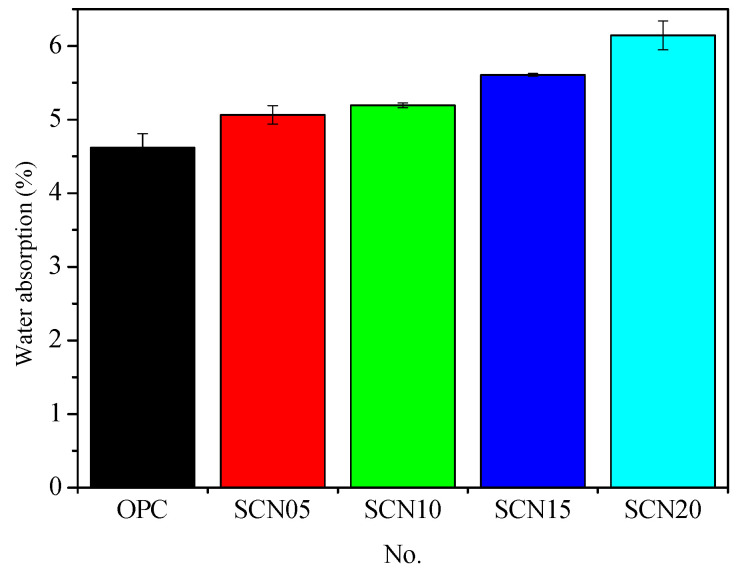
Comparison chart of water absorption rates between SCC and OPC.

**Figure 9:**
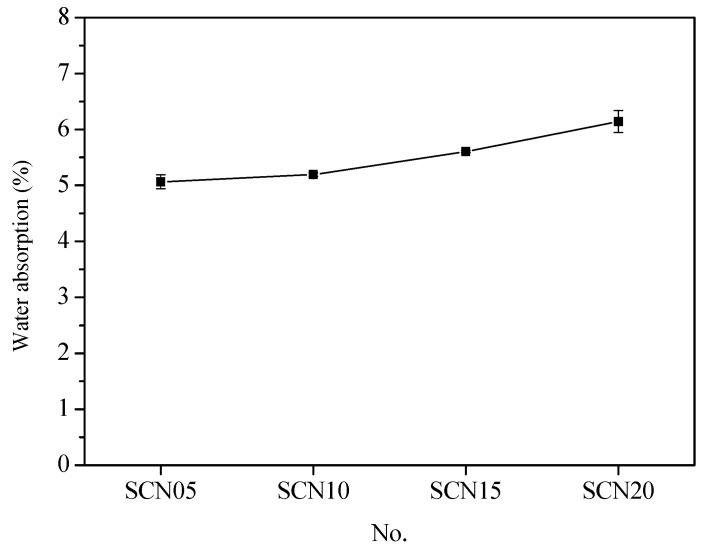
Relationship curve of water absorption rates for SCC with different NaOH addition levels.

**Figure 10:**
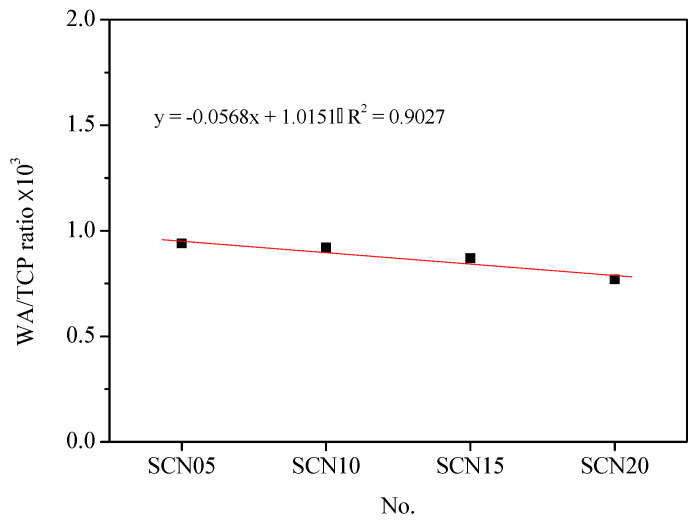
Trend chart of water absorption (WA) and cumulative total charge passed (TCP) in SCC.

Figure 8 gives information about the water absorption rate comparison between OPC and SCC. The results showed that SCC catalyzed by different NaOH concentrations had higher rates than OPC. Particularly, at 20% NaOH concentration, the water absorption rate was highest (about 32.87%). The water absorption rates measured about 9.52% for SCN05, around 12.34% in SCN10, and nearly 21.43% for SCN15. The best performance was observed at 0.5% NaOH concentration. This implies that any incremental change in the alkaline substance concentration led to a rise in the cement pores. In other words, the water rate extension indicated the poor performance of cement (Figure 9). Furthermore, Figure 10 shows the linear relationship between water absorption rates and cumulative total charge passed with a slightly downward trend (R_2_ = 0.90). This emphasized the decrease in SCC durability.

### 3.4. Impact of Alkali–Aggregate Reaction on the Durability of SCC

**Figure 11:**
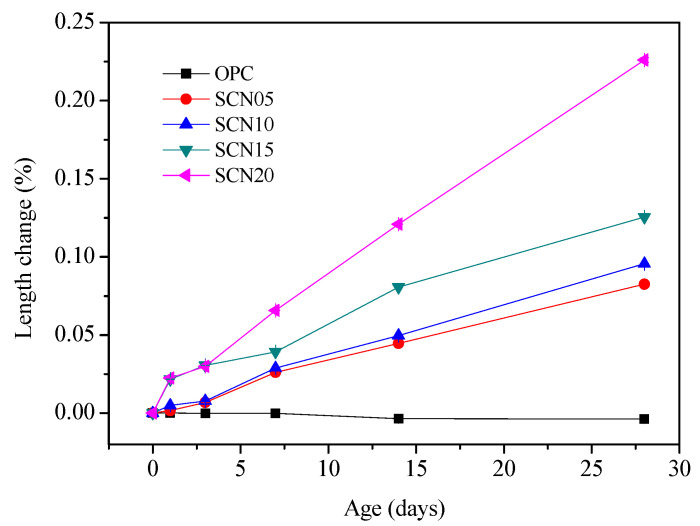
Volume change curves for different curing ages of SCC and OPC.

**Figure 12:**
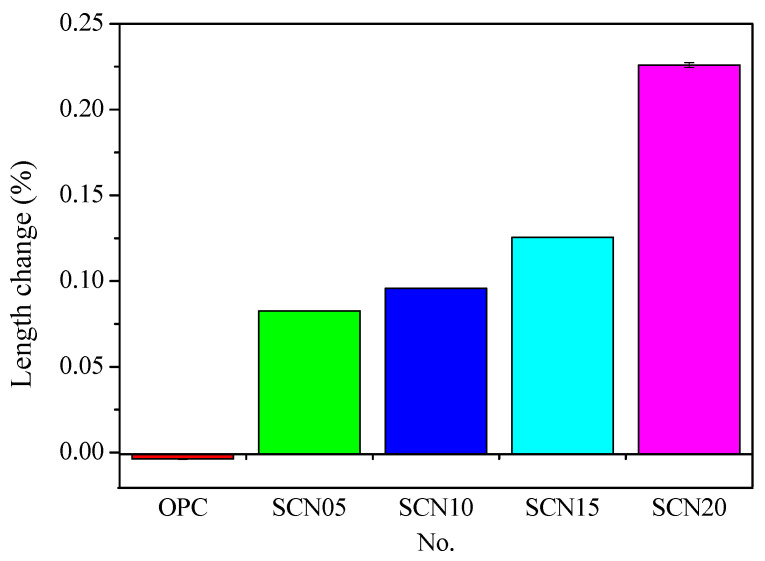
Comparison of volume change between SCC and OPC after 28 days of curing.

Figure 11 provides information about the volume variations of SCC and OPC over different curing ages. Overall, there is a rising trend in the length of SCC at various NaOH concentrations. The volume of OPC slightly decreased with increasing curing age, while the SCC trend changed dramatically at different concentrations of NaOH. A considerable change was observed in SCC length at 20% NaOH concentration. The length variations after 28 days of curing showed a slight difference in expansion between SCN05 and SCN10. In contrast, SCN15 and SCN20 have shown a significant increase in length (Figure 12).

**Figure 13:**
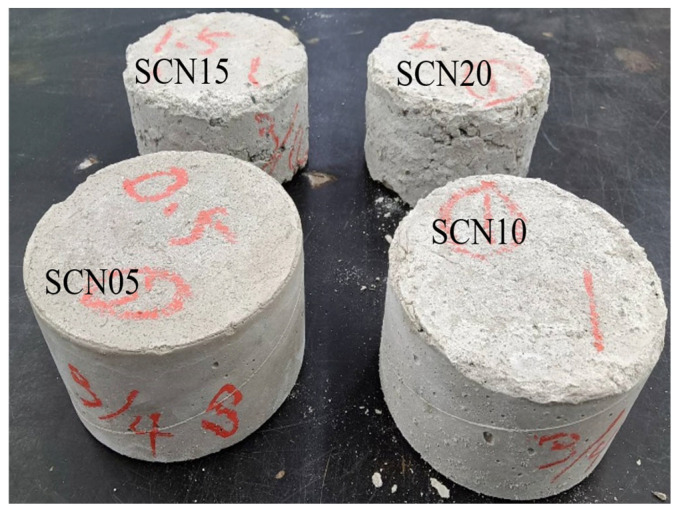
Appearance of hardened SCC with different NaOH addition levels.

**Figure 14:**
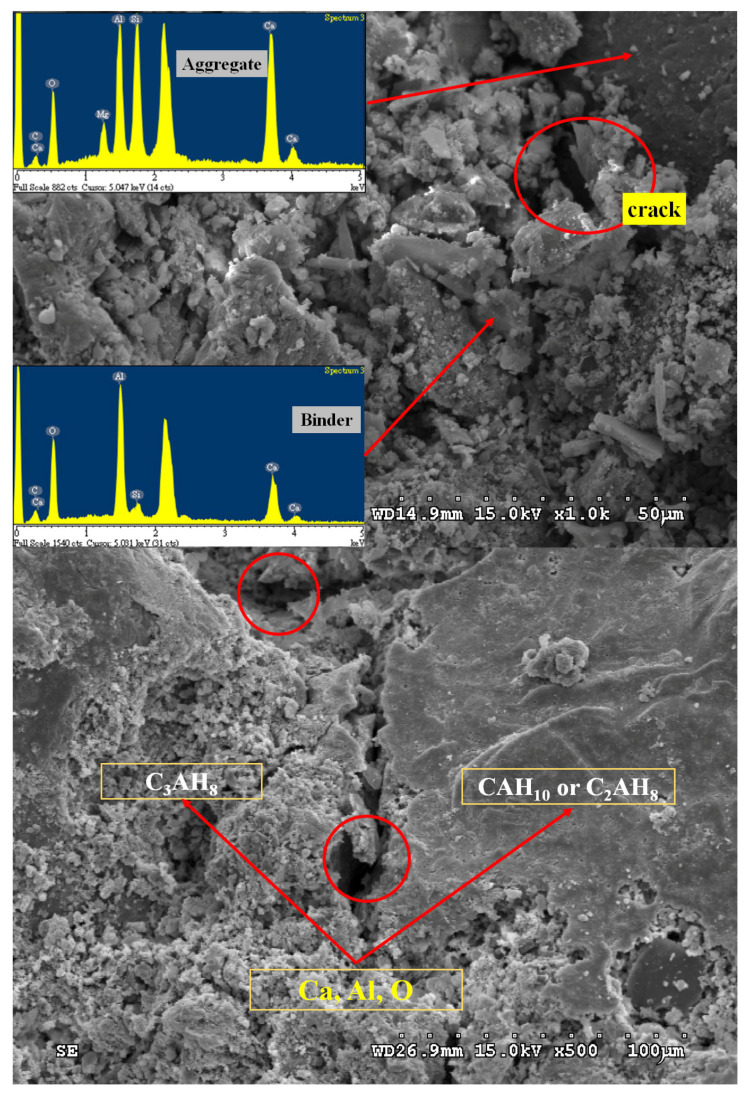
Microstructure of SCC with NaOH addition level of 2.0%.

Figure 13 also shows that SCC with higher NaOH dosages has pop-outs and, in some cases, surface cracking or fissures. SEM microstructure analysis emphasized the presence of surface cracks (Figure 14). Therefore, it is assumed that the volume expansion in SCC can be attributed to the SCC expansion volume being intensified by the higher amount of NaOH. The main reason is the alkali–aggregate reaction between the NaOH catalyst and natural aggregates, which impacts SCC durability. Accordingly, in terms of durability, it is recommended that if NaOH is utilized as a catalyst, it should not exceed 0.5% of the weight ratio of the solid content of the SC.

The findings revealed a clear relationship between NaOH dosage and the durability of SCC. The length variation in SCC specimens increased progressively with higher NaOH concentrations, which indicated internal expansion during the curing period. This expansion was more obvious at NaOH dosages of 1.5% and above (SCN_15_ and SCN_20_), where significant length increases and visible surface distress, such as pop-outs and cracking, were observed (Figure 13).

Alkalis originating from aggregates have been identified as a key factor in the degradation of calcium aluminate cement (CAC)-based concrete. Alkaline hydrolysis and alkali–silica reactions (ASR) commonly begin at or near the surface of the concrete, leading to the formation of loosely bound bayerite (Al(OH)_3_) deposits. These deposits compromise the bond between the aggregates and the cementitious matrix, ultimately causing a marked decline in mechanical strength [31].

Scanning electron microscopy (SEM) analysis confirmed the presence of internal fissures. Figure 14 shows the microstructure of SCC with a magnification of 1000 times. This supported the hypothesis that elevated NaOH content accelerates deleterious chemical reactions within the cement matrix. EDS analysis of the microstructure of the SCC sample showed that the cement matrix mainly contained Ca, Al, and O, which is consistent with the typical hydration products of high-alumina cement, such as CAH_10_, C_2_AH_8_, and C_3_AH_6_. However, in the aggregate region, originally expected to consist primarily of SiO_2_, other elements were also detected, such as Ca, Al, Si, and O. This suggested chemical interactions, which indicated that the primary mechanism behind the observed expansion was alkali–silica reaction (ASR). In the presence of NaOH, reactive silica in the aggregate reacted with the alkaline solution to form expansive alkali–silica gel. The gel absorbed moisture and swelled, which generated internal tensile stresses. This process led to microcracking and surface deterioration. The severity of this phenomenon increased with higher alkali content. Therefore, the pronounced cracking was observed in SCC mixtures with NaOH dosages above 0.5%, indicating that the alkaline aggregates were responsible for the cracking. Although a 0.5% NaOH dosage (SCN05) caused slight expansion compared to OPC, no visible macrocracks were detected at this level. This threshold serves as a reference to mitigate AAR-induced expansion and cracking risk in SC. It is suggested that even more conservative dosages and extended durability monitoring be used for applications requiring higher structural reliability or long-term performance under challenging environmental conditions. The results indicated that structural deterioration mainly arises from two key mechanisms. The first involves the transformation of metastable hydration products, such as CAH₁₀ and C_2_AH_8_, into the more stable phase C_3_AH_6_ under environmental influences. This phase transition leads to a further loosening microstructure and increased porosity. The second mechanism is the alkali–silica reaction (ASR), which causes volumetric expansion, cracking, and progressive degradation of the microstructure.

### 3.5. Influence of SC Composition on Compressive Strength

**Figure 15:**
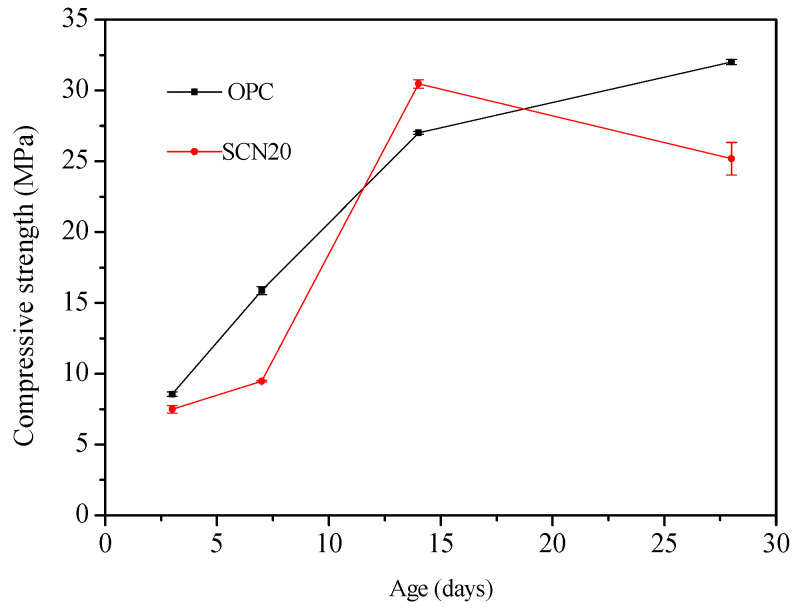
Relationship between curing ages and compressive strength of SCC and OPC.

Compressive strength is a key indicator of concrete durability. SCN20 was selected to explore the impact of NaOH addition on the compressive strength of SCC compared to OPC, as this sample showed the most significant change in strength over time. This made it particularly suitable for illustrating the durability challenges associated with liquid cement concrete. The compressive strength of OPC steadily increased with curing age, which is typical for conventional cement. SCN20 followed a similar trend initially, and its compressive strength was already close to OPC at 14 days. However, a noticeable decreasing trend was observed in the compressive strength of SCN20 after 15 days (Figure 15).

Table 3 shows the chemical composition of SC and Portland cement (CEM I). The primary components of SC are Al_2_O_3_ (50.3%) and CaO (40.28%), which show characteristics of high-alumina cement. In contrast, CEM I consists of CaO (62.82%) and SiO_2_ (20.44%), highlighting a clear compositional difference between SC and OPC. The compressive strength development of CEM I is mainly related to the hydration of C_2_S and C_3_S, which gradually form C-S-H gel over time and enhance the strength of the cement. However, in high-alumina cement, the mechanism is different. Hydration of C_3_A initially forms metastable phases such as CAH_10_ and C_2_AH_8_. Over time and under the influence of temperature and humidity, these metastable phases transform into a more stable phase, 3CaO·Al_2_O_3_·6H_2_O (C_3_AH_6_). This process leads to the formation of numerous pores. Therefore, this transformation increases the porosity of the matrix, which can adversely affect long-term strength.

**Figure 16:**
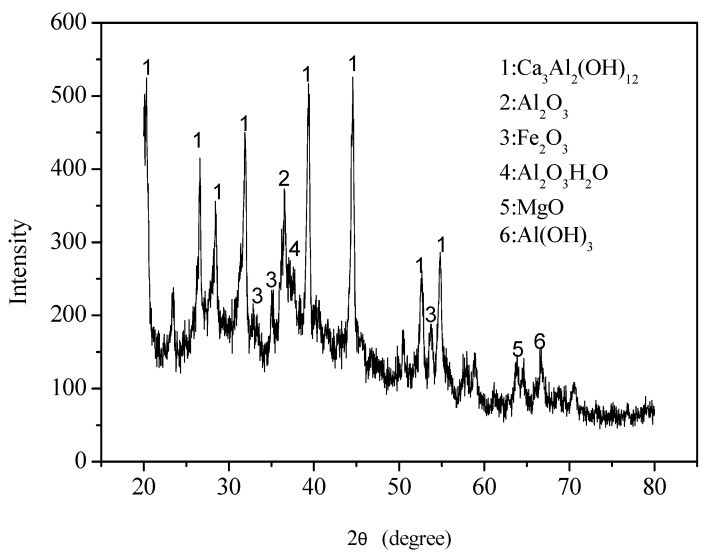
Phase structure of the SC sample cured for 28 days.

In addition, the XRD analysis of hardened SC after 28 days of curing (Figure 16) showed the presence of several crystalline phases, including Ca_3_Al_2_(OH)_12_, Al_2_O_3_, Fe_2_O_3_, AlO(OH)·H_2_O, MgO, and Al(OH)_3_. The highest intensity peaks suggest dominant phases, particularly those labeled “1”, indicating a significant presence of Ca_2_Al_2_(OH)_12_. The pattern reveals a complex mixture of crystalline phases. These phases reinforced the hypothesis that the reduction in compressive strength observed at 28 days in SCC was attributed to chemical phase transformations. Moreover, the error bars for SCN20 at 28 days show noticeable variation, indicating a degree of instability (Figure 15). This variability may be linked to the formation of internal microcracks, potentially caused by alkali–aggregate reactions within the SCC matrix (as illustrated in Figure 14). This microstructural damage can lead to inconsistencies in compressive strength measurements.

### 3.6. Influence of pH on the Durability of SCC

**Figure 17:**
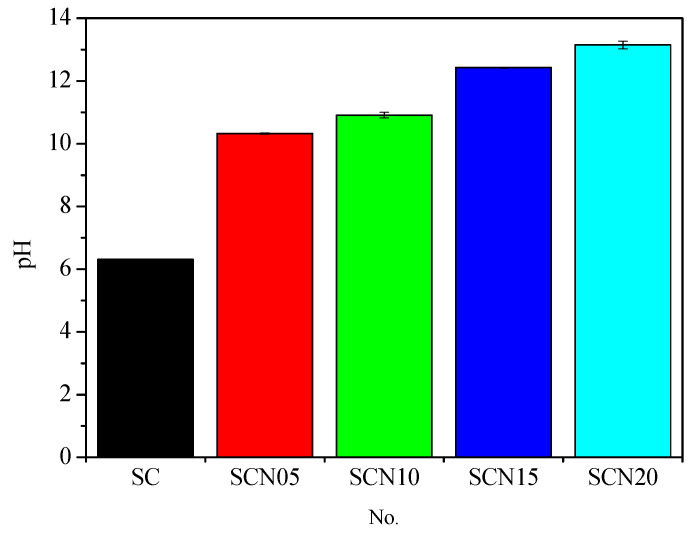
pH variation of SC and SCC after NaOH addition.

Figure 17 illustrates the pH variation in SC and SCC with NaOH concentrations ranging from 0.5% to 2%. The results showed that the addition of 0.5% NaOH led to a substantial increase in pH, rising from an initial value of 6.31 to 10.32. Further increases in NaOH concentration to 1%, 1.5%, and 2% resulted in corresponding pH elevations to 10.92, 12.23, and 13.15, respectively. Previous studies have shown that the elevated alkalinity, resulting from the presence of Na⁺ and K⁺ ions, significantly enhances the risk of alkali–silica reaction (ASR) [35]. Furthermore, as illustrated in Figure 3 and Figure 12, both the cumulative electrical charge passed and the length expansion of the specimens increased proportionally with the NaOH content. This trend suggests enhanced ionic mobility and the development of internal stresses, both of which contribute to ASR-induced deterioration. In this study, NaOH was used as a catalyst to enhance the pH and reactivity of the SC. However, the resulting increase in alkalinity also induced expansion and microcracking, which negatively impacted the long-term durability of the concrete. Therefore, it is recommended that the NaOH dosage be limited to below 0.5% to achieve an optimal balance between activation efficiency and durability performance.

## 4. Conclusions

According to the experimental results of this study, the findings are summarized as follows:

1. The best performance was observed at 0.5% NaOH concentration, which indicated better durability of SCC. However, increasing the dosage of NaOH has led to a decrease in SCC durability. RCPT results showed an increase in the cumulative total charge passed of SCN05 of about 0.84%, which is almost similar to OPC. This was also confirmed by the salt pond test.
2. There was a noticeable correlation between the rise in alkali concentration and the water absorption rate of SCC. Furthermore, the linear relationship between water absorption rate and cumulative total charge passed indicated that the primary factor influencing SCC durability is less porosity.
3. NaOH causes volume expansion in SCC, and the expansion becomes more pronounced with an increase in NaOH dosage. The primary reason for volume expansion is the alkali–aggregate reaction resulting from the interaction between the NaOH catalyst and natural aggregates. Therefore, in terms of durability, it is recommended for SCC, if NaOH is used as a catalyst, not to exceed 0.5% of the weight ratio of the SC’s solid content.
4. The incorporation of NaOH successfully initiated the hydration process of SC, allowing it to demonstrate characteristics typical of high-alumina cement. However, after 28 days, a decrease in strength and structural stability was observed, likely due to phase transformations commonly associated with high-alumina cement and potential alkali–aggregate reactions. These factors may significantly contribute to the reduced long-term durability of SCC.

Although SC shows significant potential as a low-carbon alternative with versatile application possibilities, its structural application is still in the early stages of research. There are several limitations related to SC. First, SC requires an alkaline catalyst for activation, which can trigger alkali–aggregate reactions if not properly controlled. The second limitation is the durability uncertainty of SC, due to the lack of long-term field data and limited durability investigation under varying environmental conditions. Third, the compressive strength of Sc can decline over time due to phase transformations from metastable (CAH_10_, C_2_AH_8_) to stable phases (C_3_AH_6_). This increases porosity and compromises mechanical integrity. Finally, at higher alkali dosages, internal expansion may lead to microcracking, which poses concerns for the structural reliability of SC in load-bearing applications. These challenges require further research to optimize SC formulation and improve its performance for long-term structural applications.

This study mainly focused on the influence of NaOH catalyst on the durability of the material, with porosity assessed indirectly through water absorption to evaluate overall performance. It is suggested that future research incorporate advanced pore structure characterization techniques to better understand how the NaOH catalyst influences pore structure at the micro- and nanoscale. This would provide a more detailed interpretation of the mechanisms governing corrosion and durability in alkali-activated materials. Moreover, future studies are encouraged to include phase composition analyses and long-term durability assessments to gain deeper insight into the chemical stability and performance of the material over time.

## Figures and Tables

**Table 1:** Mixture proportions (kg/m^3^).

No.	Water	Cement I	SC	Coarse Aggregate	Fine Aggregate	NaOH
OPC	200	312.50	-	1035.00	719.20	
SCN05	-	-	512.50	1035.00	719.20	1.53
SCN10	-	-	512.50	1035.00	719.20	3.13
SCN15	-	-	512.50	1035.00	719.20	4.69
SCN20	-	-	512.50	1035.00	719.20	6.25

**Table 2:** Test results.

Test Item No.			OPC	SCN05	SCN10	SCN15	SCN20
**RCPT**	Total charge passed (c)		5355	5400	5625	6480	8010
**Water absorption test**	Water absorption (%)		4.62	5.06	5.19	5.61	6.14
**Salt ponding test**	Chloride content (%)	0~1 cm	0.0155	0.0385	0.0482	0.0679	0.0934
		1~2 cm	0.0122	0.0372	0.0476	0.0521	0.0583
		2~3 cm	0.0036	0.0063	0.0108	0.0398	0.0517
**Length change test**	Length change (%)	1 day	0	0.0017	0.0050	0.0217	0.0225
		3 days	−0.0001	0.0068	0.0078	0.0306	0.0300
		7 days	−0.0001	0.0261	0.0288	0.0392	0.0658
		14 days	−0.0035	0.0446	0.0496	0.0806	0.1210
		28 days	−0.0038	0.0826	0.0957	0.1255	0.2260

**Table 3:** Analysis of SC and cement composition.

Chemical Composition	SC (wt%)	CEM I (wt%)
**Na_2_O**	6.0	-
**MgO**	1.1	3.07
**Al_2_O_3_**	50.30	5.18
**SiO_2_**	0.92	20.44
**P_2_O_5_**	0.86	-
**K_2_O**	0.12	-
**CaO**	40.28	62.82
**Fe_2_O_3_**	0.13	3.12
**SO_3_**	-	2.26
**Others**	0.29	3.11

## Data Availability

The original contributions presented in the study are included in the article, further inquiries can be directed to the corresponding author.

## Abbreviations

The following abbreviations are used in this manuscript:

AAR	Alkali–aggregate reaction
AASHTO T259	Standard Method of Test for Resistance of Concrete to Chloride Ion Penetration
ASTM C114–18	Standard Test Methods for Chemical Analysis of Hydraulic Cement
ASTM C1202–19	Standard Test Method for Electrical Indication of Concrete’s Ability to Resist Chloride Ion Penetration
ASTM C157–75	Standard Test Method for Length Change of Hardened Cement Mortar and Concrete
C2S	Dicalcium silicate
C3S	Tricalcium silicate
CA	Calcium aluminate
CO2	Carbon dioxide
K2O	Potassium oxide
KOH	Potassium hydroxide
LCAC	Liquid calcium aluminate cement
Na2O	Sodium oxide
NaOH	Sodium hydroxide
OPC	Ordinary Portland cement
RCPT	Rapid chloride penetration test
SC	Suspended Cement
SCC	SC-based concrete
SEM	Scanning electron microscopy
XRD	X-ray Diffraction
